# Supplementary material for: Long-term results of hypofractionation with concomitant boost in patients with early breast cancer: A prospective study
Source: PLoS One. 2021 Oct 7;16(10):e0258186. doi: 10.1371/journal.pone.0258186 (PMC8496829; doi:10.1371/journal.pone.0258186)
Supplement: Attachment — Submitted filename: response to reviewers.docx [file pone.0258186.s001.docx]

**Response to reviewers**

**Reviewer #1**

I think that this topic should be related to toxicity and not to efficacy.

However, the Authors could explain the reason to expect an impact with the sib on efficacy: they could talk about the BED in the Materials&Methods and not in the discussion.

Another important topic to underline is the boost: i believe that more

details about the patient' age should be analyzed because of the different impact on efficacy in relation the the age. Because of one of the benefit of the hypofractionation with SIB is the reduction of overall treatment time, this data must be reported in the results section.

Moreover, data about toxicity are mandatory, as shown in the

introduction section (line 54-58): could you describe the tolerance of

your patients?

**Response:**

I have added the BED in the Materials and Methods section.

I have added the late toxicity information.

I have added the age and boost information in the patient characteristic.

The Authors stated that patients are T1-3 N0-1 (line 73), but this doesn't reflect what they said at line 79.

**Response:**

I have changed the sentence of patient characteristic that included in my study.

How did you calculate the follow up? Since surgery? The end of RT?

Please explain

**Response**:

I calculated follow up since surgery.

Could You describe the follow up of your patients? Did the Surgeon or

The Radiation Oncologist follow the patients?

**Response**:

I have added the follow up section which explain that the patients were followed up by surgeons, radiation oncologists and medical oncologists.

How did you make the clinical diagnosis of the recurrence?

**Response:**

We did the imaging (CT scans) and the tissue biopsy.

What kind of baseline characteristics did you collect?(i.e.:comorbidities, tumor biology, ki 67…ecc...)

**Response:**

I have added the patient characteristic in table 1.

The total number of the patients is low: why did the patients decline to take part in the study? You said that both radiotherapy fractionation schemes are standard of care…

**Response:**

We calculated the number of patients by using match pair method, with a 90% of power. Pearson Chi-square test was used for testing the difference between groups with an alpha value of 0.05.

RESULTS

line 120: please 68.5 % should be reported in letters because at the beginning of the sentence.

**Response:**

I have changed it to be the letters.

The baseline characteristics should be expanded with tumor characteristics

(biology, KI 67, lymphovascular invasion...): this could justify (or not) the low number of recurrence

**Response:** I have added the information of baseline characteristics.

-A stratification by age sould as follows should be reported: patients aged ≤40 years, 41–50 years, 51–60 years, >60 years.

**Response:**

I have done it in the table 1.

In the section of "outcomes" you have to report both with absolute number and % (not only one). Moreover the recurrence must be describe in details: i don't understand the value of 17.8% (what kind of recurrence? global?). If more details are shown with the absolute number, it could be easier to understand the results.

**Response**:

I have added the absolute outcome.

In table 2, patient n 35 received 54.4Gy/16 fr, but this fractionation doesn't correspond to your hypofractionation of 43.2 in 16 fraction with a sib of 0.6 Gy for each fraction: please explain.

**Response:**

I am sorry it is wrong number.

I understand that the events number are low, but you could report the Kaplan-Meier curves because your follow-up is very long.

**Response:**

I have added the graph in the paper.

DISCUSSION

This section should explain the impact of the SIB on efficacy outcomes:

you should expand what you've reported at lines 160-163. please, correct line 17 (remove "In" at the the beginning).

Finally, at line 181 you introduced toxicity, but this is not a topic of your

study: i believe that this is the most importanty aspect to explore in the

setting of hypofractionation with SIB. Please expand.

**Response:**

I have added the toxicity information.

**Reviewer #2**

Major criticisms:

1. Materials and methods, Study population: the Authors report that they

enrolled patients treated from 2005 to 2009 in the study, while in the

abstract they refer to the period between October 2009 and June 2010.

Please correct it.

**Response:** I have corrected the period time to be from 2009 to 2010

2. Materials and methods, Study population: “The eligibility criteria

included patients aged 18 or older, with resected tumour margins wider

than 1 mm, with no lymph node involvement”. But on page 4, line 73,

the Authors described to enrolled “all patients with operable invasive

breast cancer (T1-3N0-1M0)...”.

I suggest to better define the eligible criteria for patients included in this

analysis.

**Response:** I have corrected the eligibility of patients to be

Eligibility criteria included patients with operable invasive breast cancer

(T1-3N0-1M0), aged 18 or older with resected tumor margins wider than

1 mm.

3. Materials and methods: how was the follow-up performed?

(surveillance time, radiological examinations, etc.)

**Response:** I have added the follow up examinations

Follow up examinations

Patients were followed up every 3-4 months for the first few years and 6 months for year 4 and 5 and then yearly. They were seen by surgeons, radiation oncologist and medical oncologist in the separate clinic. At each follow-up, medical history, physical examination and toxicity were performed and assessed. Mammogram and breast ultrasound was performed 6 months after radiation therapy and then yearly.

4. On page 4, line 77, the Authors state that “assignment of patients to

each arm was made at the discretion of the treating radiation oncologist”. This statement is inaccurate and I suggest to better define the assignment criteria in the two treatment schedules. This is the weakest point of this study - SELECTION BIAS.

**Response:** I would add this as the limitation of the study. But we found that the baseline characteristics of both groups are comparable of the two groups. I have changed the statement to be the treatment schedules were assigned by the radiation oncologists.

5. In the results section the reported baseline characteristics are not clear (for example from lines 119 to 120 the Authors reported that “71.2% of the patients were ER positive and 24.7% were ER negative”, but the sum of the percentages is not 100%). I suggest to add a table with patient

characteristics and their correct percentages.

**Response:** I added patient characteristics in the table 1

For the ER status of the patients: 71.2 % was ER positive and 24.7% was ER negative. The rest of the patients were unknown.

6 Results: the Authors described the different schedules of adjuvant chemotherapy. But, from line 121 to 124, they wrote “All patients received one of the following adjuvant chemotherapy...”, while in table 1 we can see that not all patients were treated with chemotherapy. Please correct this information.

**Response**: I have corrected this sentence

For those patients who received adjuvant chemotherapy, they receive

one of the following regimens

7. The study focused on survival outcomes. Taking into account the long follow-up, it would be interesting to report data regarding treatment related toxicity. If available, I suggest to add it.

**Response:** I have added the information of late toxicity outcome.

Minor limitations

1. Table 1: reported number of patients and percentages approximation are wrong. For examples in stage and treatment sections the number of patients underwent conventional or hypofractionated treatment are 39 and 34, respectively.

**Response**: I have added the table of patient characteristic.

2. I suggest to add the pathological and biological characteristics in table

1.

Response: I have added those information in table 1

3. In the outcomes section (from line 141 to 142): reported rates of

recurrence and metastasis are not clear.

**Response:** I have added and clarified the information.

4. Line 146: reported percentages refer to 10-year OS for Conv-SEQ is

different from that described in the abstract. Please correct it.

**Response:** I have corrected the number in the abstract to be 91.6% vs 91.9%.

5. Table 2: all abbreviations and acronyms should be explained at first use. DISH was not previously defined.

**Response**: I have explained all abbreviations
