# Supplementary material for: Long-term results of hypofractionation with concomitant boost in patients with early breast cancer: A prospective study
Source: PLoS One. 2021 Oct 7;16(10):e0258186. doi: 10.1371/journal.pone.0258186 (PMC8496829; doi:10.1371/journal.pone.0258186)
Supplement: Attachment — Submitted filename: Response to reviewers.docx [file pone.0258186.s002.docx]

**Response to reviewers**

**Reviewer #4**

**INTRODUCTION**

1) Line 49. The statement “The conventional radiation to the whole breast is 45–50 Gy, administered in 1.8–2 Gy per fraction doses over 5–6 weeks” needs bibliographic reference.

**Response:** I have added the references which are

-Fisher B, Anderson S, Bryant J, et al. Twenty-year follow-up of a randomized trial comparing total mastectomy, lumpectomy, and lumpectomy plus irradiation for the treatment of invasive breast can- cer. N Engl J Med 2002;347:1233-41.

- Veronesi U, Luini A, Del Vecchio M, et al. Radiotherapy after breast-preserving surgery in women with localized cancer of the breast. N Engl J Med 1993;328:1587- 91.

2) Line 53. I suggest to add reference FAST Trial doi: 10.1200/JCO.19.02750. Epub 2020 Jul 14.

**Response:** I have added FAST trial reference.

3) I recommend adding the data from Krug's recent article. “Adjuvant hypofractionated radiotherapy with simultaneous integrated boost after breast-conserving surgery: results of a prospective trial” <https://doi.org/10.1007/s00066-020-01689-7>

**Response:** Thank you, I have added the data from Krug’ article as you suggested.

**MATERIALS**

1) Line 99. The paragraph “Radiotherapy” is lacking. I suggest to add the data of the Linac used.

**Response:** I have added the data regarding our treatment machines.

2) Line 103. “The details of the radiation technique have been described previously. [(21])” The reference provided describes a concomitant boost, not a simultaneous boost. I suggest to better describe the technique used and specify whether it is a concomitant or simultaneous boost. Although many authors use the 2 terms indistinctly, they are different. Concomitant boost treatment involves performing 2 different plans in the same fraction. Simultaneous boost treatment involves performing a single plane through intensity-modulated techniques.

**Response:** Thank you for the comment. Our study, we use concomitant boost and I have put more detailed about the technique in this paper. I also added the treatment planning image.

3) I suggest to add contouring information and treatment plan images.

**Response:** I have added the image. (The 3D planning) There are whole breast and electron boost planning.

**RESULTS**

1) Collecting dosimetric data and correlating them with recurrence or skin toxicity could consolidate the study.

**Response:** We really appreciate your suggestion. Unfortunately, 60% of our patients were treated with 2D techniques. As a result, the collection of dosimetric data may not be complete. However, we made every effort to make it as prescribed in the radiation technique. For whole breast irradiation, photons 6 MV with a source-to-axis distance of 100 cm were used in all patients. Dose homogeneity of breast was achieved by using wedges. For tumor bed boost, an en-face electron beam with appropriate energy was used, prescribing at the 90% isodose line.

**DISCUSSION**

1) Line 190. Several studies described use concomitant boost, but you refer to simultaneous boost. I recommend changing the terms.

**Response:** I have changed it to be a concomitant boost.

2) I suggest extending the discussion by analysing the advantages and disadvantages of 2D and 3D versus intensity-modulated techniques. I would recommend a few references on this topic.

**Response:** Thank you very much for all the references. I have added those related information in the manuscript.

3) I recommend emphasising the importance of this study in the perspective of the Covid-19 pandemic period.

**Response:** I have added the sentence that this paper could be used in the context of the Covid-19 situation.

**CONCLUSION**

1) Also in the conclusion and thus in the title, you should better define whether it is a concurrent or simultaneous boost.

**Response:** I have changed into the concomitant boost.

**Reviewer 5#**

the topic is interesting due to a lack from randomized studies. Unfortunately, even if your effort is remarkable, the sample size of the population, the retrospective nature of your study and the absence of statistical approach to increase the homogeneity of the population (pooled analysis or similar) are strong limitations. Furthermore some part of the paper are not written in a fluent English and somewhere grammatical errors are present and this lowered the quality of the paper.

**Response:** We are grateful for your comment. Also there is the limitation of the study, we would like to report our long-term clinical and toxicity data which is the key strength of our study. This study will support the use of hypofractionation with concomitant boost in breast cancer. We also submit our manuscript for English editing in order to improve the quality of the paper.
